# Three-Dimensional Printed Teeth in Endodontics: A New Protocol for Microcomputed Tomography Studies

**DOI:** 10.3390/ma17081899

**Published:** 2024-04-19

**Authors:** Tiago Reis, Cláudia Barbosa, Margarida Franco, Ruben Silva, Nuno Alves, Pablo Castelo-Baz, Jose Martín-Cruces, Benjamín Martín-Biedma

**Affiliations:** 1 Endodontics and Restorative Dentistry Unit, School of Medicine and Dentistry, University of Santiago de Compostela, 15701 Santiago de Compostela, Spain; pepe3214@gmail.com; 2 Centre for Rapid and Sustainable Product Development (CDRSP), Polytechnic University of Leiria, 2411-901 Leiria, Portugal; margarida.franco@ipleiria.pt (M.F.); ruben.j.silva@ipleiria.pt (R.S.); nuno.alves@ipleiria.pt (N.A.); 3 FP-I3ID, FP-BHS, Health Sciences Faculty, University Fernando Pessoa, 4249-004 Porto, Portugal; cbarbosa@ufp.edu.pt; 4 RISE-Health, University Fernando Pessoa, 4249-004 Porto, Portugal; 5 Oral Sciences Research Group, Endodontics and Restorative Dentistry Unit, School of Medicine and Dentistry, University of Santiago de Compostela, Health Research Institute of Santiago de Compostela (IDIS), 15706 Santiago de Compostela, Spain; pablocastelobaz@hotmail.com (P.C.-B.); benjamin.martin@usc.es (B.M.-B.)

**Keywords:** endodontics, three-dimensional printed teeth, PolyJet, support material, microcomputed tomography

## Abstract

This study aimed to describe a support material removal protocol (SMRP) from inside the root canals of three-dimensional printed teeth (3DPT) obtained by the microcomputed tomography (microCT) of a natural tooth (NT), evaluate its effectiveness by comparing the 3DPT to NT in terms of internal anatomy and behaviour toward endodontic preparation, and evaluate if 3DPT are adequate to assess the differences between two preparation systems. After the SMRP, twenty 3DPT printed by PolyJet™ were microCT scanned before preparation and thereafter randomly assigned into two groups (n = 10). One group and NT were prepared using ProTaper Gold^®^ (PTG), and the other group with Endogal^®^ (ENDG). MicroCT scans were carried out after preparation, and the volume increase, volume of dentin removed, centroids, transportation, and unprepared areas were compared. For the parameters evaluated, no significant differences were found between the 3DPT and NT before and after preparation (*p* > 0.05), and no significant differences were found between the 3DPT PTG group and the 3DPT ENDG group (*p* > 0.05). It can be concluded that the SMRP described is effective in removing the support material SUP706B™. PolyJet™ is adequate for printing 3DPT. Furthermore, 3DPT printed with high-temperature RGD525™ have similar behaviour during endodontic preparation with PTG as the NT, and 3DPT can be used to compare two preparation systems.

## 1. Introduction

The American Association of Endodontists defines root canal preparation as “Procedures involved in cleaning and shaping the canal system prior to obturation”, distinguishing between “biomechanical preparation” as the “use of rotary/reciprocating and/or hand instruments to expose, clean, enlarge and shape the pulp canal space, usually in conjunction with irrigants” and “chemomechanical preparation” as the “use of chemicals for irrigation of the root canal, demineralization of dentin, dissolution of pulp tissue and neutralization of bacterial products and toxins; used in conjunction with biomechanical preparation” [1].

Over the last few decades, root canal preparation protocols have changed, and many new nickel–titanium systems have become available; nonetheless, clinicians require knowledge of shaping properties and a performance evaluation of these systems to select them according to clinical cases [2]. Root canal preparation ex vivo studies provide useful and valuable data to improve the biological outcome of preparation and therefore have to be continued in the future [3].

Extracted human natural teeth (NT) are considered the gold standard for ex vivo studies; however, they present several disadvantages, the main one being very difficult standardization [4], not only as a consequence of the root canal system anatomy but also the donor’s age, which has an influence on the dentin properties [5,6]. Three-dimensional printed teeth (3DPT) obtained through the microcomputed tomography (microCT) of real NT have been used as an alternative for NT in both research and teaching and offer a good opportunity to create balanced experimental groups [3,7,8,9,10,11,12,13,14]. The major concern expressed about 3DPT is the difference in radiopacity and hardness between resin and human dentin [3,7,9,10,12]. Nonetheless, protocols for the standardization of studies using 3DPT still have to be developed [3].

PolyJet^®^ (Stratasys Ltd., Eden Prairie, MN, USA) printing is based on layer-by-layer technology. The process consists of the nozzles of the printer moving along the XY plane and spraying liquid photosensitive resin on the printer bed, and a UV lamp cures the resin. After the first layer is finished, the printer bed will drop by a layer thickness in the Z plane, and the deposition of another layer is repeated. This is a potentially attractive option for low-volume manufacturing in research environments. Where hollow parts or overhangs exist, the nozzles spray a layer of removable support material [15,16,17]. Support material removal methods include manual breaking, dissolution under water pressure or with sodium hydroxide (NaOH), or melting [15,18,19]. However, it has been reported that there is difficulty in removing the support material inside 3DPT root canals [3,7,8,17,20], so canals could be filled partially or totally with support material, which may have an effect on microCT analyses before and/or after preparation [21].

In order for 3DPT to be used in ex vivo preparation studies, their internal anatomy should be similar to the original NT, they should be support-material-free, their behaviour during preparation should be identical to that of NT, and they should be capable of assessing the differences between two preparation systems. To the authors’ knowledge, there are no studies that describe a protocol for removing the support material from inside the root canals of 3DPT printed by PolyJet™. In addition, the available literature regarding the printing accuracy of internal anatomy is still scarce.

Therefore, the first aim of this study was to evaluate the effectiveness of a support material removal protocol (SMRP) for inside root canals by comparing the internal anatomy of a NT with 3DPT. The null hypothesis was that there was no difference between the internal anatomy of NT and 3DPT. The second aim of this study was to compare the behaviour under preparation with ProTaper Gold^®^ (Dentsply-Sirona, Fair Lawn, NJ, USA) (PTG) between NT and 3DPT. The null hypothesis was that there was no difference in preparation behaviour between NT and 3DPT. The third aim of this study was to compare the behaviour of 3DPT during preparation with two different preparation systems, namely PTG and Endogal^®^ (Endogal, Galician Endodontics Company, Lugo, Spain) (ENDG). The null hypothesis was that no difference existed between PTG and ENDG.

## 2. Materials and Methods

The study protocol was approved by the ethics committee of Fernando Pessoa University (FCS/PI 429/23). Based on data from a previous study [22] in which shaping ability was assessed, a sample size calculation was performed using G*Power 3.1.9.7 software for Windows (Heinrich Heine, Universität Düsseldorf, Düsseldorf, Germany) with an α type error of 0.05 and a β power of 0.95 for an effect size of 1.79 input into the *t* test family, resulting in a required sample size of 16 samples (8 per group) to observe significant differences between groups. Ten samples were used per group to compensate for possible sample loss during experimental procedures.

### 2.1. Natural Specimen Selection

**Figure 1:**
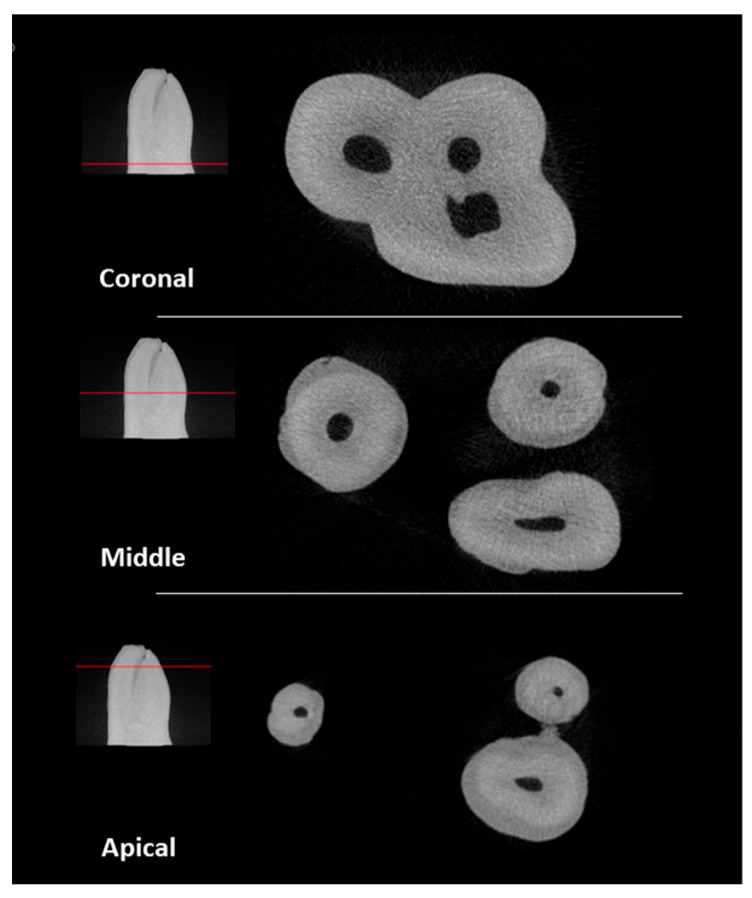
Cross-sectional views of microCT scan of the natural tooth.

An initial pool of 45 maxillary permanent molars, extracted for reasons unrelated to this study, was used. The teeth were collected and stored in distilled water until use. Radiographs were taken in mesiodistal and buccolingual directions to ensure that the inclusion criteria were met. The inclusion criteria were teeth with fully formed apices, the absence of root fractures, no signs of external and internal resorption or decayed tissue in the region of interest, the absence of previous endodontic treatment, and a degree of curvature between 20° and 40°. The degree of curvature was measured according to Schneider’s method [23]. From the initial sample, 14 teeth were selected. The endodontic cavities were prepared using a round diamond bur #4 and an Endo-Z™ bur (Dentsply Sirona, Fair Lawn, NJ, USA) driven by a high-speed handpiece under water cooling. The canals were explored with a K-file #10 (Dentsply Sirona) until the tip of the file was just visible through the apical foramen to ensure the existence of canal patency. The tooth crowns were sectioned at 4 mm from the cementoenamel junction to create a platform for easing future references. The specimens were then scanned using a microCT device (Skyscan 1174; Bruker, Kontich, Belgium) at 50 KV and 800 mA energy; a 0.25 mm thick aluminium filter was used, with rotational steps of 1.0 increments for a total rotation of 180°, a 16.65 μm image pixel size, and an exposure time of 12,000 ms. Images were reconstructed using NRecon version 1.7.46 software (Bruker, Kontich, Belgium, in which algorithms were introduced for the correction of ring artefacts (3), smoothing (3), and beam hardening (40%). CTAn version 1.20.3.0 software (Bruker, Kontich, Belgium) was applied to produce one STL file of each tooth and another of the respective canal’s anatomy. The STL files were exported to a free software platform (MeshLab version 2021.10) for qualitative canal configuration evaluation and the election of the NT to replicate. The tooth chosen was a second left maxillary molar that presented 3 fully separated roots, each one with a single independent canal, without any lateral canals. The mesiobuccal root presented an oval canal with a degree of curvature of 32°; the distobuccal root presented a round small diameter canal with a degree of curvature of 30°; and the palatal root presented a round large diameter canal with a degree of curvature of 26° (Figure 1).

**Figure 2:**
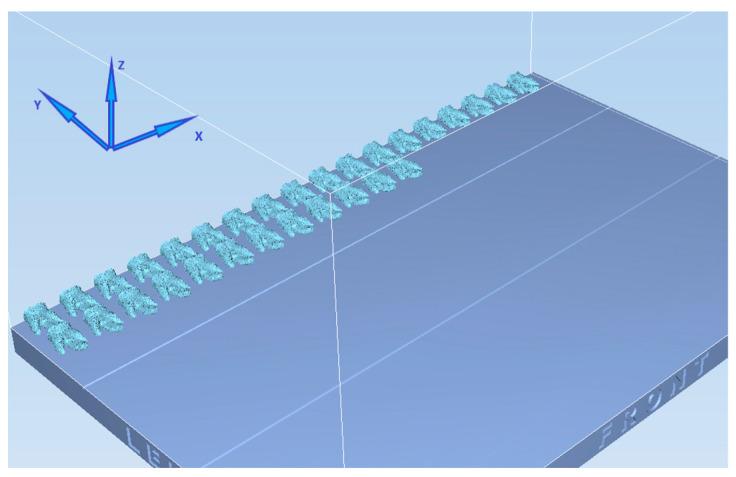
Teeth orientation on the printer bed.

The STL file of the tooth was simplified and prepared for 3D printing using the “Simplification: Quadric Edge Collapse Decimation” filter [14] because a high-resolution STL file is an excessively large file that 3D printing softwares has difficulties processing [4]. The original STL file presented 3,298,498 vertices and 6,598,466 faces with a 314 Mbytes size, and the simplified STL presented 798,881 vertices and 1,599,232 faces with a 76.2 Mbytes size. The STL file was printed by PolyJet™ using a Stratasys Object30 Prime™ printer (Stratasys Ltd., Eden Prairie, MN, USA) in high-quality mode with a layer thickness of 16 µm. High-temperature RGD525™ (Stratasys Ltd.) and the support material SUP706B™ (Stratasys Ltd.) were the materials chosen for printing. The tooth was displayed horizontally with the mesial surface in contact with the printer bed, with its long axis parallel to the X axis of the printer head and printer bed (Figure 2).

**Figure 3:**
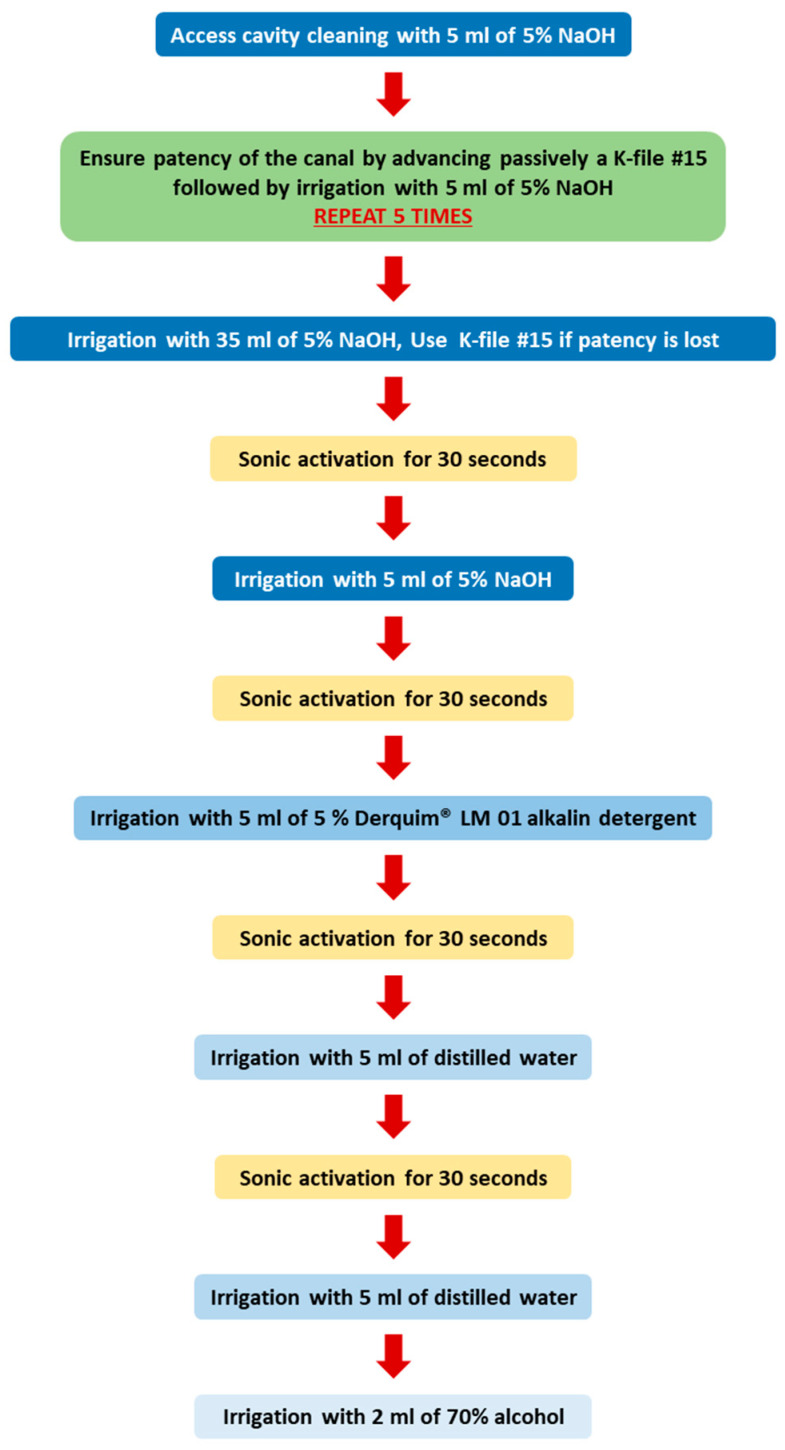
Flowchart of support material removal protocol.

After printing, the support material involving the teeth and inside the access cavity was removed manually. The support material removal protocol (SMRP), summarized in a flowchart (Figure 3), involved using a K-file #15 (Dentsply Sirona), a 30G polypropylene body needle Irriflex^®^ (Produits dentaires SA, Vevey, Switzerland), and an Endoactivator^®^ (Dentsply Sirona) with a small Endoactivator^®^ Tip (15/0.02) (Dentsply Sirona). Irrigants were used, namely a 5% solution of NaOH, 5% Derquim^®^ LM 01 alkalin detergent (ITW Reagents, S.R.L., Castellar del Vallès, Spain), distilled water, and 70% alcohol. The SMRP steps were (1) access cavity cleaning with 5 mL of 5% NaOH, and for each canal, (2) advance passively into the canal the K-file #15 until the tip of the file is just visible through the apical foramen to ensure the patency of the canal, followed by irrigation with 5 mL of 5% NaOH at high pressure. This step was repeated 5 times to achieve irrigant extrusion at the end of this step. Then, it was followed by (3) irrigation with 35 mL of 5% NaOH; during this step, if irrigant extrusion was lost, meaning losing the patency, a K-file #15 was used as before; (4) irrigant sonic activation with an Endoactivator^®^ (Dentsply Sirona) at a high frequency for 30 s with up-and-down movements, with an amplitude of 4 mm; (5) irrigation with 5 mL of 5% NaOH; (6) irrigant sonic activation as described before; (7) irrigation with 5 mL of 5% Derquim^®^ LM 01 alkalin detergent; (8) irrigant sonic activation as described before; (9) irrigation with 5 mL of distilled water; (10) irrigant sonic activation as described before; (11) irrigation with 5 mL of distilled water; and (12) irrigation with 2 mL of 70% alcohol.

**Figure 4:**
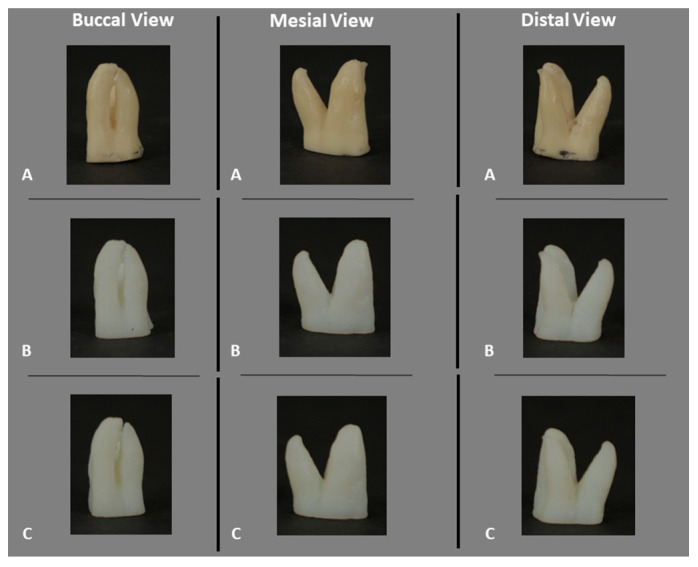
Natural teeth (**A**) and 3D-printed teeth (**B**,**C**) from different views.

In this way, a 20-3DPT sample (n = 20) randomly assigned (www.random.org, accessed on 10 January 2023) to each of the two experimental groups (n = 10), PTG and ENDG (Figure 4), was obtained.

In sequence, the 3DPT were scanned using a microCT device (Skyscan 1174; Bruker) at 50 KV; 800 mA of energy; and rotational steps of 1.0 increments for a total rotation of 180° with a 16.65 μm image pixel size and an exposure time of 12,000 ms. The images were reconstructed using NRecon v 1.7.46 software, in which algorithms were introduced for the correction of ring artefacts (5), smoothing (3), and beam hardening (50%).

### 2.2. Root Canal Preparation

All the preparations were performed by a single operator with 22 years of clinical experience in the field of endodontics and previous experience in using PTG and ENDG systems clinically. The teeth were mounted in place using the ProTrain system^®^ (Simit Dental Srl, Mantua, Italy). The working length (WL) was determined by taking 1 mm from the value obtained during the SMRP. The WL was 16 mm for the buccal canals and 15 mm for the palatal canal. An electric motor X-Smart^®^ Plus (Dentsply Sirona) was used to operate the files with in-and-out pecking motion (2–3 mm amplitude) in a continuous clockwise rotation according to the manufacturer’s recommendations.

### 2.3. Preparation of NT and Group I with PTG

A glide path was created by using a ProGlider^®^ instrument (Dentsply Sirona) (a size tip of 16 and progressive taper from 0.02 to 0.08) until the WL was reached. All files from the PTG system, which have a convex triangular cross section and progressive taper [24], were used up to the WL except the SX file, which was used only for coronal interference removal, in the sequence SX (19/0.04), S1 (18/0.02), S2 (20/0.04), F1 (20/0.07), and F2 (25/0.08). Patency was checked after the use of each instrument with K-file #10. The instruments were used for one tooth preparation; after that, they were discharged. Root canal irrigation was performed between each file with 5 mL of 5.25% NaOCl (Cerkamed, Stalowa Wola, Poland) for NT and 5 mL of distilled water for 3DPT using an Irriflex^®^ (Produits dentaires SA, Vevey, Switzerland) needle and activated with an Endoactivator^®^ at a high frequency with a small tip for 30 s. After preparation, the canals were irrigated two times with 5 mL of irrigant and activated with the Endoactivator^®^ (Dentsply Sirona), so the total volume of irrigation for each canal was 40 mL. In the end, the canals were dried with paper points (Dentsply Sirona).

### 2.4. Preparation of Group II with ENDG

A glide path was created by using the A (15/0.03) instrument until the WL was reached. All files from the ENDG system, which is a new system with a parallelogram cross section and instruments of constant 4% and 6% taper [25], were used up to the WL, except the X file, which was used only for coronal interference removal, in the sequence X (25/0.09), B (20/0.04), C (25/0.04), and D (25/0.06). Patency was checked after the use of each instrument with K-file #10. The instruments were used for one tooth preparation; after that, they were discharged. Root canal irrigation was performed between each file with 5 ml of distilled water using an Irriflex^®^ needle and activated with the Endoactivator^®^ at a high frequency with a small tip for 30 s. After preparation, the canals were irrigated three times with 5 mL of irrigant and activated with Endoactivator^®^, so the total volume of irrigation for each canal was 40 mL. In the end, the canals were dried with paper points.

A new microCT scan was performed on NT and both 3DPT groups according to the same scanning and reconstruction parameters as those established initially, respectively.

### 2.5. MicroCT Evaluation

A microCT evaluation was conducted by one of the authors, blinded to the groups. Images before and after preparation were superimposed with the 3D registration application of the DataViewer v 1.5.6.2 software (Bruker microCT), and the data obtained were processed using CTAnv v 1.20.3.0 software (Bruker microCT). The region of interest was set from the furcation region to the apex of the root.

The volume of dentine removed and centroids were quantified by subtracting the values before preparation from the values after preparation [3]. In accordance with the orientation in which the samples were evaluated, a positive value for the alteration of centroid X meant an alteration in the buccal direction, centroid Y in the mesial direction, and centroid Z in the apical direction [26]. The transportation was investigated by calculating the vectorial translocation of all sections’ X, Y, and Z coordinate values using the following formula, where “a” is after and “b” is before preparation: $\sqrt{{\left(\mathrm{Xa}-\mathrm{Xb}\right)}^{2}+{\left(\mathrm{Ya}-\mathrm{Yb}\right)}^{2}+{\left(\mathrm{Za}-\mathrm{Zb}\right)}^{2}}$ [27].

The percentage of unprepared area was calculated by the number of static voxels compared with the total number of voxels present on the root canal surface [26]. The tooth volume expansion evaluated for the 3DPT corresponds to the ratio of the 3DPT tooth volume compared to the NT volume [13].

### 2.6. Statistical Analysis

The collected data were processed using IBM SPSS Statistics version 26.0 software. The Shapiro–Wilk test was applied to verify data normality. Accordingly, for normal and non-normal distributions, a one-sample *t*-test or one-sample Wilcoxon signed-rank test and an independent-sample *t*-test or Mann–Whitney U test were applied. The significance level was 5% for all statistical tests (*p* < 0.05).

## 3. Results

**Figure 5:**
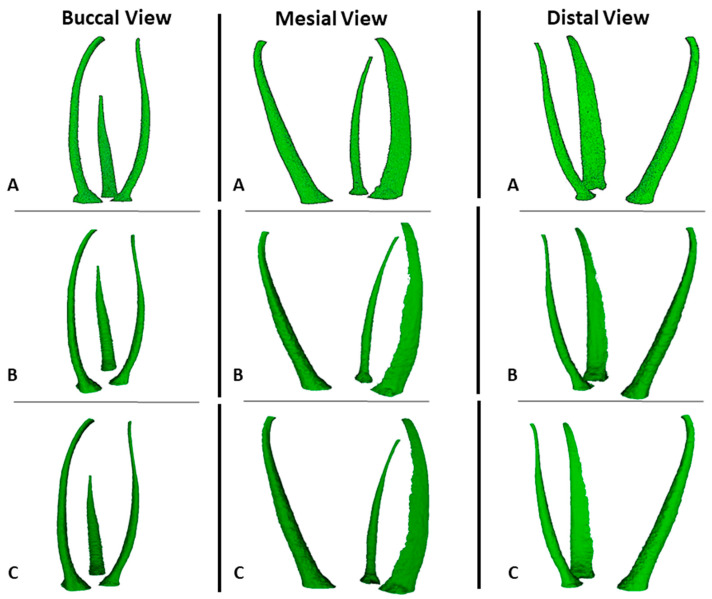
Representative 3D reconstruction of microCT scans after the support material removal protocol and before preparation from different views. (**A**) natural tooth; (**B**) 3D-printed teeth by ProTaper Gold^®^ group; and (**C**) 3D-printed teeth by Endogal^®^ group.

**Figure 6:**
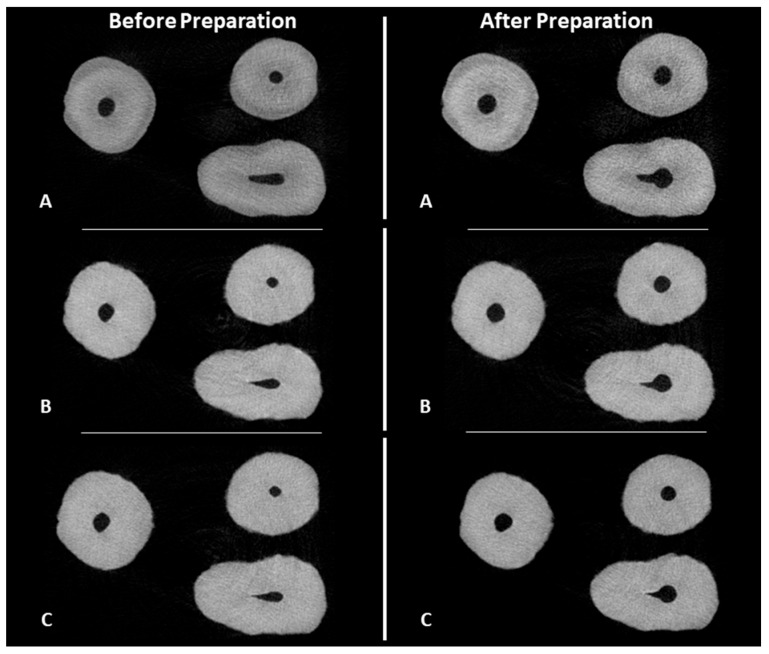
Cross-sectional views of microCT scans before and after preparation. (**A**) natural tooth; (**B**) 3D-printed teeth with ProTaper Gold^®^ group; and (**C**) 3D-printed teeth with Endogal^®^ group.

The measurements of canal volume, centroid X, Y, and Z before preparation, and the percentage of tooth volume expansion for the 3DPT are shown in Table 1. There were no statistically significant differences between the NT and the 3DPT for all the variables (*p* > 0.05) (Figure 5 and Figure 6).

**Figure 7:**
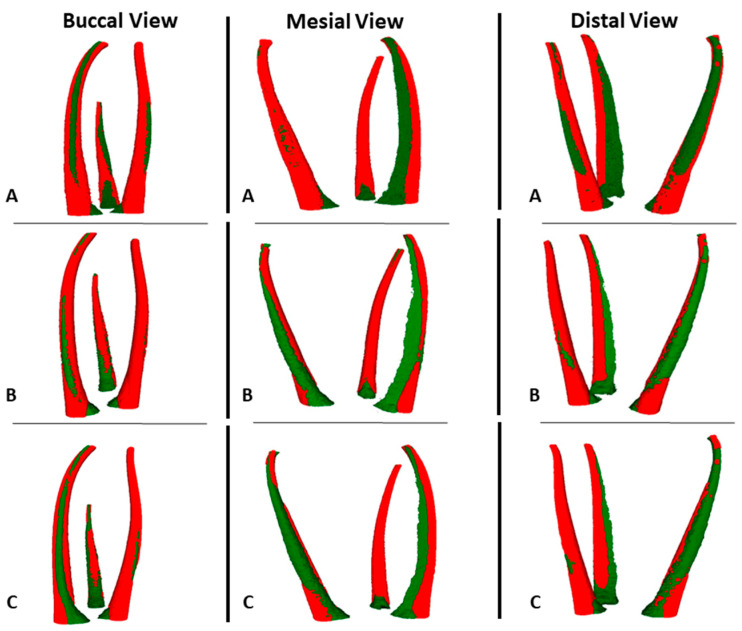
Representative 3D reconstruction of microCT scans before (green) and after (red) preparation from different views. (**A**) natural tooth; (**B**) 3D-printed teeth with ProTaper Gold^®^ group; and (**C**) 3D-printed teeth with Endogal^®^ group.

A comparison between the NT and 3DPT before and after preparation with the PTG system is presented in Table 2. There were no statistically significant differences between the NT and the 3DPT for all the variables before preparation (*p* > 0.05). The 3DPT showed a similar behaviour to the NT after preparation since there were no statistically significant differences (*p* > 0.05) related to the measurements of canal volume, centroid X, Y, and Z after preparation, the volume of dentin removed, the percentage of volume increase, centroid X, Y, and Z alteration, transportation, and the percentage of unprepared areas (Figure 6 and Figure 7).

The overall results of the measurements before preparation, as well as the results of the shaping performance of the PTG and ENDG instrument systems on the 3DPT, are presented in Table 3. There were no statistically significant differences between the two groups for all the variables before and after preparation (*p* > 0.05). (Figure 6 and Figure 7).

## 4. Discussion

The present study described an SMRP and compared the root canal anatomy between a NT and 3DPT based on a microCT scan of the NT sample. Comparing the volume and centroids X, Y, and Z of the NT and the 3DPT before preparation, our findings demonstrated no statistically significant differences between them. Therefore, the null hypothesis was accepted. These findings show that the SMRP described is effective in removing the support material SUP706B™ used in this study, and PolyJet™ is adequate for printing 3DPT with similar internal anatomy to NT. The SMRP described here is based on the generic manufacturers’ indications for removing the support material SUP706B™ by solubilization, in which an alkaline 2% solution of NaOH and a 1% solution of sodium metasilicate are used in a cleaning station, followed by water rinsing [19]. NaOH is the basis of the SMRP described here, and Derquim^®^ LM 01 alkalin detergent was used, which is a detergent based on NaOH and anionic and non-ionic surfactants, to exert cleaning action on the root canal walls to mimic the detergent function of sodium metasilicate.

Sonic activation with an Endoactivator^®^ was used to improve the irrigants’ action by producing intra-canal irrigant agitation and streaming. Since the contact of the Endoactivator^®^ tip with the root canal walls inhibits its free oscillation, reducing irrigant streaming, the tip was placed as deeply as possible without contacting the walls [28,29]. Nevertheless, even if contact occurs, the Endoactivator^®^ tip is a polyamide, which does not produce active root canal cutting, diminishing the risk of root canal anatomy alteration [30]. The Irriflex^®^ needle used is a polyethylene flexible needle that has a smoother progression inside the canal and does not wedge against root canal walls. It presents two side openings that produce two jets oriented in the direction of the root canal walls and delivers a large volume of irrigant at a high flow rate with a clinically minimal risk of apical extrusion [31]. However, for the SMRP to be effective, the irrigation must be carried out at high pressure and with the needle tip as close to the apex as possible for irrigant extrusion to occur.

Nevertheless, our findings show differences in the values of the 3DPT before the instrumentation of the parameters evaluated, meaning that the 3DPT are not equal between them. Relative to the root canal anatomy, this could be a result of our protocol since canal patency is mandatory for its effectiveness, being traduced, as stated before, in the visual observation of the extrusion of the irrigants. K-file #15 was chosen for this purpose since in channels under 150 µm, it is very difficult to remove the support material [17], and it was used obligatorily five times for each canal. After this number of utilizations, if the patency was lost, K-file #15 was used to reestablish it. Although used in a passive way, this means that the number of K-file #15 insertions was not the same for all the canals, and its effects on the root canal anatomy should be considered [20,32].

In relation to the total volume of the 3DPT, the alterations in volume that occur with PolyJet™ printing in order to expand have been described [33,34]. The findings of this study show that there is a 0.77% volume expansion, and this can also explain the differences between the 3DPT. The result of the present study is comparable to the 0.71% referred to in a previous study [13]; however, in that study, the STL file of the 3DPT and the volumetric analyses were realised by a 3D scanner in contrast with the microCT methodology of the present study.

Another issue that should be addressed is that, although PolyJet™ has the smallest dimensional error compared to other 3D printing technologies [16,35,36,37], its accuracy depends on the material used, the geometry of the printed object, and the orientation given in relation to both the printer head and the printer bed [15,34,38]. In a previous study, a rectangular flat part was printed with the long axis parallel to the X, Y, or Z axis of the printer bed, and it was concluded that the long axis of the printed part should be parallel to the X axis [38]. However, to the authors’ knowledge, this feature was never studied while printing teeth, and in a single root tooth, the long axis would have been easily defined as the long axis of the root. In the present study, a multiple-root tooth was used, so it was measured as a whole, and the longest distance was in the buccal-to-palatal direction, so this was considered the long axis, and the 3DPT were printed as shown in Figure 2. Nevertheless, future studies should assess which is the best orientation to produce a multiple-root tooth according to the X, Y, or Z axis of the printer bed and if this has relevance to the accuracy of 3DPT. Also, it should be noted that the STL file was simplified and prepared for 3D printing, and this action may also have resulted in some level of distortion [13]. Future studies should assess which level of simplification is supported by the STL file without significant distortion relative to NT.

The NT used in the present study had its crown sectioned and presented an internal anatomy with three single different types of canals; however, it did not present any anatomical irregularities such as lateral canals or isthmus, which are known to be only accessible to irrigants [39]. Future studies are needed to evaluate the effectiveness of the support material in teeth with a full crown and with anatomical irregularities.

Regarding the second aim of this study, in order for 3DPT to be used in ex vivo preparation studies, their behaviour during preparation should be like NT. The present results regarding the volume of dentin removed, the percentage of volume increase, transportation, and unprepared areas show that there were no statistically significant differences between them. Therefore, the null hypothesis was accepted, and it can be concluded that 3DPT behave in a similar way to the NT when prepared with PTG. The major concern expressed about 3DPT is the difference in hardness between resin and human dentin [3,7,9,10,12]. High-temperature RGD525™ is an opaque model material that has exceptional dimensional stability and presents a tensile strength of 70–80 Mpa, a modulus of elasticity of 3200–3500 Mpa, and a flexural strength of 110–130 MPa [40]. In comparison, human dentin presents a tensile strength of 44.4–97.8 MPa, with a lower value for the inner dentin near to the pulp [41], a modulus of elasticity of 1375–1931 MPa [42], and a flexural strength of 171–254 MPa [43]. All these values between the printing material and dentin are approximated; however, it should be noticed that the material structure differs from the tubular structure of dentin.

Regarding the third aim, the results for the volume of dentin removed, the percentage of volume increase, transportation, and unprepared areas show that there were no statistically significant differences between the two preparation systems. So, the null hypothesis was accepted. Nevertheless, although there are no statistically significant differences, it is observed that ENDG produces a lower volume increase, a lower volume of dentin removed, and a higher percentage of unprepared area, thereby demonstrating that 3DPT are suitable for evaluating the differences between two preparation systems.

To the authors’ knowledge, there is no other study that evaluates, using microCT, the shaping properties of the ENDG system, so the present results cannot be compared to others. Regarding the results observed for the PTG system in the present study, the percentage of volume increase was 47.68%, and in the literature, it ranges from 18.7% to 163.32% [22,24,44,45,46]. The percentage of unprepared area was 54.97%, while in the literature, it ranges from 3.57% to 46.85% [22,24,44,45,46,47,48]. The differences between the present study results and the literature can be explained by the type of teeth or canals used. Most studies use mandibular molar mesial canals; these studies show higher values for the percentage of volume increase and smaller values for the percentage of unprepared area [22,24,45,46], compared to, for instance, studies that use, for example, mandibular incisors or premolars [44,48]. The variation in the canal geometry has an effect on the preparation techniques [49]. It is widely accepted that preparing oval-shaped canals is a challenge, and smaller values for the percentage of unprepared area in this type of canal are associated with brushing movements during preparation and not with pecking movements [50,51].

In the present study, all the preparations were carried out by an experienced dentist in the field of endodontics with clinical experience with the two systems, and the major critical comment of the operator was a higher screw-in effect in the 3DPT compared to the NT, which results in a more difficult preparation, even with a controlled pecking movement of a 2–3 mm amplitude. The screw-in effect is the tendency of a rotary instrument to be pulled into the canal. It is affected by the type of movement kinematics, the cross section, or the taper of the instrument, as well as by the rotational speed [52,53,54,55]. In the present study, the manufacturer’s recommendations of rotation per minute (rpm) and torque were used. Future studies are needed to establish how changes in rpm, torque, pecking movement amplitude values, and instrument design can influence 3DPT preparation.

As said before, protocols for the standardization of studies using 3DPT still have to be developed [3]. The present study, within its limitations, presents a description of an effective protocol for support material SUP706B™ removal, demonstrating that 3DPT printed with high-temperature RGD525™ material have similar behaviour during endodontic preparation to that of NT. Nonetheless, we propose that future research needs to achieve a standardisation of studies using 3DPT. In summary, the establishment of the optimal orientation regarding the printer bed of multiple-root teeth; the maximal level of simplification of the STL file without losing information if the SMRP described is effective in other types of root morphology; and establishing how changes in the values of the rpm, torque, and amplitude of pecking movements and instrument design can influence the preparation of 3DPT.

## 5. Conclusions

Within the limitations of the present study, it can be concluded that the SMRP described is effective in removing the support material SUP706B™. PolyJet™ is adequate for printing 3DPT. Furthermore, 3DPT printed with high-temperature RGD525™ have similar behaviour during endodontic preparation with PTG to that of NT, and 3DPT can be used when comparing two preparation systems.

## Figures and Tables

**Table 1:** Microcomputed tomographic analysis before preparation of natural teeth and 3D-printed teeth.

Data	Natural Tooth	3D-Printed Teeth	
Canal Volume (mm^3^)	12.08	Mean ± SD	11.99 ± 0.55
		Median	12.07
Centroid X (mm)	10.92	Mean ± SD	10.92 ± 0.09
		Median	10.93
Centroid Y (mm)	10.37	Mean ± SD	10.35 ± 0.07
		Median	10.35
Centroid Z (mm)	8.03	Mean ± SD	8.02 ± 0.09
		Median	8.04
Tooth Volume Expansion (%)		Mean ± SD	0.73 ± 0.77
		Median	0.68

**Table 2:** Microcomputed tomographic analysis before and after preparation with ProTaper Gold^®^ of natural tooth and 3D-printed teeth.

Data		Natural Tooth	3D-Printed Teeth	
Canal Volume (mm^3^)	Initial	12.08	Mean ± SD	12.12 ± 0.42
			Median	12.12
	After	17.75	Mean ± SD	17.89 ± 0.69
			Median	17.66
	Volume of dentin removed	5.68	Mean ± SD	5.77 ± 0.66
			Median	5.67
	% Volume increase	46.99	Mean ± SD	47.68 ± 6.09
			Median	45.50
Centroid X (mm)	Initial	10.92	Mean ± SD	10.92 ± 0.07
			Median	10.87
	After	11.38	Mean ± SD	11.33 ± 0.13
			Median	11.32
	Alteration	0.46	Mean ± SD	0.41 ± 0.17
			Median	0.41
Centroid Y (mm)	Initial	10.37	Mean ± SD	10.37 ± 0.07
			Median	10.40
	After	10.50	Mean ± SD	10.53 ± 0.04
			Median	10.53
	Alteration	0.13	Mean ± SD	0.16 ± 0.07
			Median	0.15
Centroid Z (mm)	Initial	8.03	Mean ± SD	8.01 ± 0.11
			Median	8.04
	After	8.10	Mean ± SD	8.01 ± 0.10
			Median	8.02
	Alteration	0.07	Mean ± SD	0.01 ± 0.015
			Median	0.02
Transportation (mm)		0.48	Mean ± SD	0.48 ± 0.14
			Median	0.45
Unprepared area (%)		57.64	Mean ± SD	54.97 ± 3.79
			Median	53.88

**Table 3:** Microcomputed tomographic analysis before and after preparation with ProTaper Gold^®^ and EndoGal^®^ of 3D-printed teeth.

Data			ProTaper Gold^®^	
Canal Volume (mm^3^)	Initial	Mean ± SD	12.12 ± 0.42	11.86 ± 0.66
		Median	12.12	11.82
	After	Mean ± SD	17.89 ± 0.69	17.02 ± 0.61
		Median	17.66	17.06
	Volume of dentin removed	Mean ± SD	5.77 ± 0.66	5.24 ± 1.02
		Median	5.67	5.49
	% Volume increase	Mean ± SD	47.68 ± 6.09	45.02 ± 10.89
		Median	45.50	47.25
Centroid X (mm)	Initial	Mean ± SD	10.92 ± 0.07	10.92 ± 0.11
		Median	10.87	10.96
	After	Mean ± SD	11.33 ± 0.13	11.37 ± 0.06
		Median	11.32	11.35
	Alteration	Mean ± SD	0.41 ± 0.17	0.45 ± 0.09
		Median	0.41	0.43
Centroid Y (mm)	Initial	Mean ± SD	10.37 ± 0.07	10.33 ± 0.06
		Median	10.40	10.32
	After	Mean ± SD	10.53 ± 0.04	10.44 ± 0.05
		Median	10.53	10.45
	Alteration	Mean ± SD	0.16 ± 0.07	0.11 ± 0.05
		Median	0.15	0.13
Centroid Z (mm)	Initial	Mean ± SD	8.01 ± 0.11	8.03 ± 0.07
		Median	8.04	8.01
	After	Mean ± SD	8.01 ± 0.10	8.07 ± 0.09
		Median	8.02	8.11
	Alteration	Mean ± SD	0.01 ± 0.015	0.05 ± 0.09
		Median	0.02	0.02
Transportation (mm)		Mean ± SD	0.48 ± 0.14	0.49 ± 0.11
		Median	0.45	0.47
Unprepared area (%)		Mean ± SD	54.97 ± 3.79	56.41 ± 5.11
		Median	53.88	55.39

## Data Availability

The data that support the finding of this study are available from the corresponding author upon reasonable request. The data are not publicly available due to privacy and ethical restrictions (undergoing PhD thesis).
